# Supplementary material for: High Predatory Capacity of a Novel Arthrobotrys oligospora Variety on the Ovine Gastrointestinal Nematode Haemonchus contortus (Rhabditomorpha: Trichostrongylidae)
Source: Pathogens. 2021 Jun 29;10(7):815. doi: 10.3390/pathogens10070815 (PMC8308572; doi:10.3390/pathogens10070815)
Supplement: Supplementary file 1 [file pathogens-10-00815-s001.zip › Table S1.pdf]

		Ranges			
	Strain	Long (µm)	Septum (µm)	Basal Cell - Septum (µm)	Septum - Distal Cell (µm)
1	A6	24.4-15.2	11.3-5.3	11.4-4.0	15.3-8.4
2	A12	21.6-15.6	10.8-6.7	9.1-4.4	14.7-9.5
3	A13	23.1-15.4	11.3-6.4	9.2-4.5	14.4-8.6
4	R2-1	21.9-15.0	11.6-6.5	8.8-3.8	14.6-9.1
5	R2-6	22.1-13.9	12.1-6.2	8.9-3.6	13.9-7.3
6	R2-13	30.3-16.0	14.8-7.2	15.8-6.0	16.3-8.3
7	R2-14	22.2-14.6	11.9-7.0	8.9-4.1	15.1-8.4

		Average			
	Strain	Long (µm)	Septum (µm)	Basal Cell - Septum (µm)	Septum - Distal Cell (µm)
1	A6	18.39	8.43	6.71	11.68
2	A12	18.62	8.03	6.68	11.94
3	A13	18.82	8.61	6.95	11.87
4	R2-1	18.53	9.01	6.68	11.85
5	R2-6	17.50	8.36	6.36	11.14
6	R2-13	21.36	8.16	8.94	12.42
7	R2-14	18.22	9.02	6.39	11.84

BC-S/S-DC
1.74
1.79
1.71
1.78
1.75
1.39
1.85
