# Supplementary figures and images for: High Predatory Capacity of a Novel Arthrobotrys oligospora Variety on the Ovine Gastrointestinal Nematode Haemonchus contortus (Rhabditomorpha: Trichostrongylidae)
Source: Pathogens. 2021 Jun 29;10(7):815. doi: 10.3390/pathogens10070815 (PMC8308572; doi:10.3390/pathogens10070815)

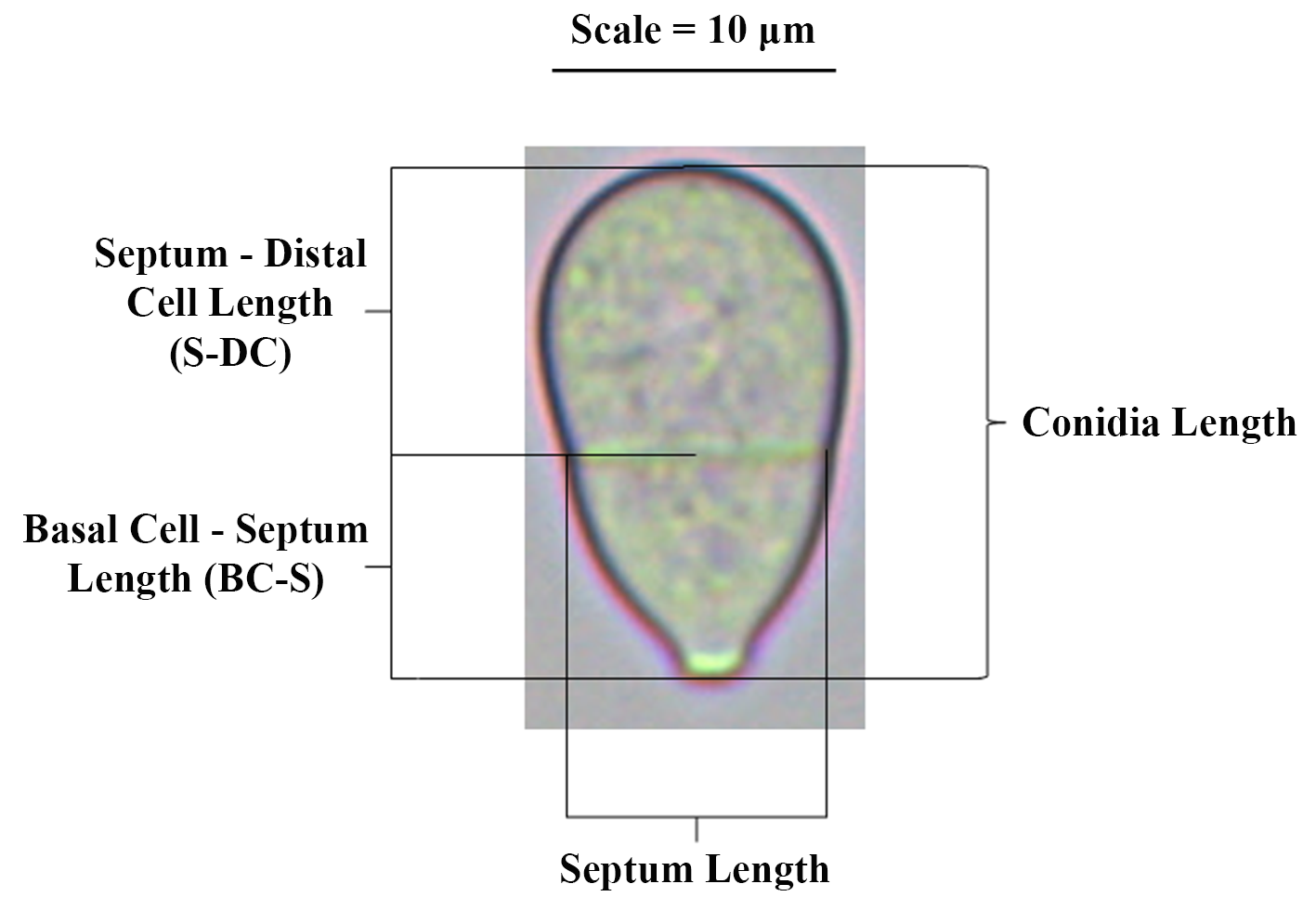

Supplement: Supplementary file 1 [file pathogens-10-00815-s001.zip › Fig. S1-2.tif]
